# Visual Sensing System to Investigate Self-Propelled Motion and Internal Color of Multiple Aqueous Droplets

**DOI:** 10.3390/s22166309

**Published:** 2022-08-22

**Authors:** Tadayoshi Aoyama, Shoki Yamada, Nobuhiko J. Suematsu, Masaru Takeuchi, Yasuhisa Hasegawa

**Affiliations:** 1 Department of Micro-Nano Mechanical Science and Engineering, Nagoya University, Nagoya 464-8601, Japan; 2 School of Interdisciplenaly Mathematical Sciences and Meiji Institute for Advanced Study of Mathemtical Sciences (MIMS), Meiji University, Tokyo 101-8301, Japan

**Keywords:** visual sensing system, self-propelled droplets, view-expansive microscope

## Abstract

This study proposes a visual sensing system to investigate the self-propelled motions of droplets. In the visual sensing of self-propelled droplets, large field-of-view and high-resolution images are both required to investigate the behaviors of multiple droplets as well as chemical reactions in the droplets. Therefore, we developed a view-expansive microscope system using a color camera head to investigate these chemical reactions; in the system, we implemented an image processing algorithm to detect the behaviors of droplets over a large field of view. We conducted motion tracking and color identification experiments on the self-propelled droplets to verify the effectiveness of the proposed system. The experimental results demonstrate that the proposed system is able to detect the location and color of each self-propelled droplet in a large-area image.

## 1. Introduction

Several studies have been conducted to reproduce the characteristics and phenomena of microorganisms using non-biomaterials to elucidate the principles underlying these microorganisms. Paxton et al. proposed the use of rod-shaped metal particles that reproduce the movements of polymastigomycotina, which move autonomously in hydrogen peroxide [1]. Ikezoe et al. developed an autonomous biochemical motor by integrating metal-organic frameworks (MOFs) with self-assembling peptides (DPAs) [2]. This micromachine artificially reproduces the chemotaxis phenomenon to control the swimming direction. Li et al. developed a micromachine incorporating a mixture of pipyridine and biphenylcarboxylic acid ligands in a UiO-67-type MOF [3]. The crystallites were metallized by metal salts to form a metal-based catalytic engine, and the micromachine had self-propelling capability. Chin et al. developed a Janus-MOF-based micromachine by crystallization of a zeolite imidazolate framework (ZIF) [4]. The Janus-MOF is produced by the epitaxial growth of ZIF-67 on the surface of ZIF-8 and crystallization. The crystals catalyze the decomposition of hydrogen peroxide on the surface of ZIF-67 and propel themselves using oxygen bubbles generated by this reaction.

In addition to the systems discussed above, there also exist self-propelled systems based on chemical droplets that contain chemical reaction schemes [5,6,7,8,9,10,11,12,13]. Wodlei et al. developed a self-propelled system consisting of dichloromethane (DCM) and the surfactant cetyltrimethylammonium bromide (CTAB) [5]. The interaction between DCM and CTAB leads to DCM evaporation, which creates a gradient in the surface tension and allows the system to move autonomously. Toyota et al. proposed self-driven droplets that exhibit self-propelled motion in aqueous dispersions of amphiphilic precursors of micrometer-sized 4-octylaniline containing 5 mol of an amphiphilic catalyst [6]. The droplets were fueled by the release of small oil droplets at their rear into the surrounding aqueous solution. Ban et al. constructed a self-driven system for surfactant-containing oil droplets that propelled them at speeds of up to 6 mm/s in the aqueous phase of a NaOH or buffer solution by a driving force based on changes in the interfacial tension due to deprotonation of the surfactant [11]. Čejková et al. compared self-driven systems of decanol droplets floating in a sodium decanoate solution with a mixture of ethyl salicylate and liquid paraffin floating in a sodium dodecyl sulfate solution to confirm similarities in the dynamics of the systems [7]. Miura et al. developed a molecular system to control the self-propelled motion of 4-heptyloxybenzaldehyde oil droplets using a gemini cationic surfactant containing carbonic bonds (2G12C) [8]. Autonomous motion occurred in the 2G12C solution of this system, indicating chemotaxis in a gradient field toward a higher concentration of sodium hydroxide. Lagzi et al. proposed a self-propelled droplet by reproducing chemotaxis with respect to the pH concentration using the chemical reactions between acids and bases and the surface tension [9]. Jin et al. constructed a self-propelled artificial swimming system that exhibits chemotaxis and negative self-motility to move autonomously by interfacial Marangoni flow induced by Micellar solubilization of the oil phase of a surfactant solution. The system was modeled using a one-dimensional diffusion process and stochastic Langevin dynamics [10]. Banno et al. devised an experimental oil-in-water emulsion system in which agglomerated particles composed of imine-containing oil transform into spherical oil droplets that first move autonomously, come to rest, and then form a film-like shape. The system was analyzed in terms of nuclear magnetic resonance (NMR), pH, and surface tension [12]. Hanczyc et al. artificially reproduced chemotaxis by adding fatty anhydride precursors to the oil phase and feeding them into a fatty acid Micellar solution to construct oil droplets that show autonomous and sustained movements through an aqueous medium [13]. Suematsu et al. constructed self-driven droplets with autonomous motion using the Belousova-Jabotinsky (BZ) reaction [14,15].

Analysis of the spontaneous motions of self-propelled droplets simultaneously requires a bird’s-eye view of multiple droplets and observations of the chemical reactions inside individual droplets. It is difficult to analyze both the motions and chemical reactions of self-propelled droplets using conventional sensing systems. Therefore, we propose a novel visual sensing system that achieves both wide-range imaging for analyzing the interactions of multiple droplets and high-resolution imaging for observation of the chemical reactions. The proposed visual sensing system is based on a view-expansive microscope [16] that can acquire both wide-range and high-resolution images. The proposed visual sensing system can detect droplets from a wide range of high-resolution images and can analyze spontaneous movements by implementing a motion tracking algorithm for view-expansive images. In addition, the system can identify the internal colors of the droplets for the analysis of chemical reactions through a color camera head and color identification algorithm. We conducted motion tracking and color identification experiments on self-propelled droplets to verify the analytical capabilities of the proposed system.

## 2. Proposed System

### 2.1. System Configuration

**Figure 1:**
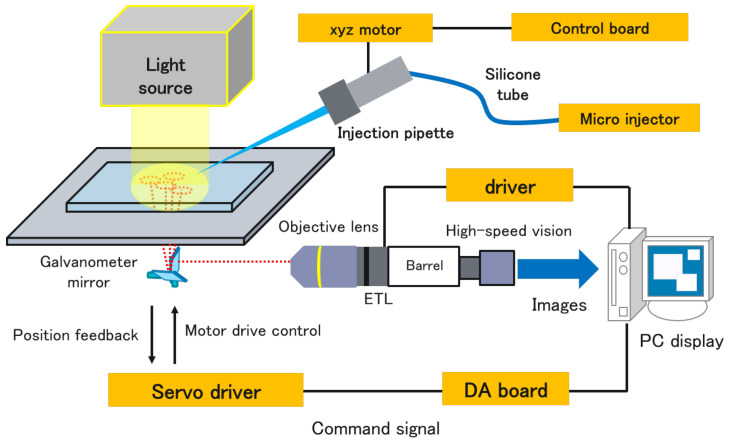
Configuration of the proposed system.

**Figure 2:**
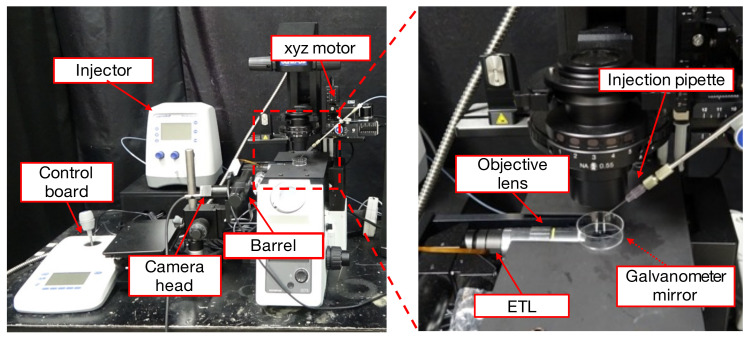
Overview of the proposed system.

Figure 1 and Figure 2, respectively, show the configuration and an overview of the proposed system. This system consists of an inverted microscope (IX73, OLYMPUS, Tokyo, Japan), a simple microscope unit (KTL-K24C-1, Kyowa Kagaku, Ishikawa Prefecture, Japan), an objective lens (LWD95 mm 10X, Kyowa Kogaku, Sagamihara, Japan), a high-speed camera (MQ003CG-CM, Ximea, Münster, Germany), a dual-axis galvanometer mirror (6210HSM 6 mm 532 nm, Novanta, Bedford, MA, USA), a variable-focus lens (Fast Electrically Tunable Lens EL 10-30-C-VIS-LD-MV, Optotune, Dietikon, Switzerland), a control computer (Windows 7 Professional 64-bit OS, HPZ440 Workstation, Intel (R) Xeon (R) CPU E5-1630 v4 3.70GHz, RAM 16.0-GB memory, HP Japan, Tokyo, Japan), a D/A board PCX-340416 (interface) optical source (high brightness 375 W metal halide, NPI, Tokyo, Japan), a microinjector (FemtoJet 4i, Eppendorf, Hamburg, Germany), and a micromanipulator (TransferMan4r, Eppendorf, Hamburg, Germany). The system acquires a wide range of images without changes in the resolution by sequentially moving the viewpoint through controlling the mirror angle and capturing multiple viewpoints. Since the proposed system combines multiple images taken with an ordinary objective lens, distortion is not a problem in the wide-range images. The pixel resolution of the system is 1 μm. In this study, we set the imaging range of the wide-range images to an elliptical region of 3.8 × 2.9 mm (8.66 mm${}^{2}$), and the frame rate is set to 1.0 frame per second (fps) by considering that the observation area is a rectangular area of 2.1 × 1.6 mm (3.36 mm${}^{2}$) and the maximum velocity of the self-propelled droplets is approximately 150 μm/s in [14] to obtain the droplets’ trajectories with sufficient time density for motion analysis. Since the pixel resolution in [14] is 10 μm, our system can observe self-propelled droplets with 10 times higher pixel resolution and the approximately 2.6 times larger area.

The proposed system has a trade-off relationship between the observable range and the frame rate. In other words, increasing the frame rate decreases the observable range, and increasing the observable range decreases the frame rate. Therefore, it is effective for high-resolution imaging of self-propelled droplets moving over a wide area at low speed.

### 2.2. Implemented Algorithm

In the proposed system, we implemented the following algorithm to detect droplets from a wide range of high-resolution images and record the trajectories of the spontaneous motions of the droplets.

(1) Acquire view-expansive image 

To acquire view-expansive images, multiple images from multiple viewpoints are captured simultaneously, similar to that reported in a previous study [16].

(2) Gamma correction 

**Figure 3:**
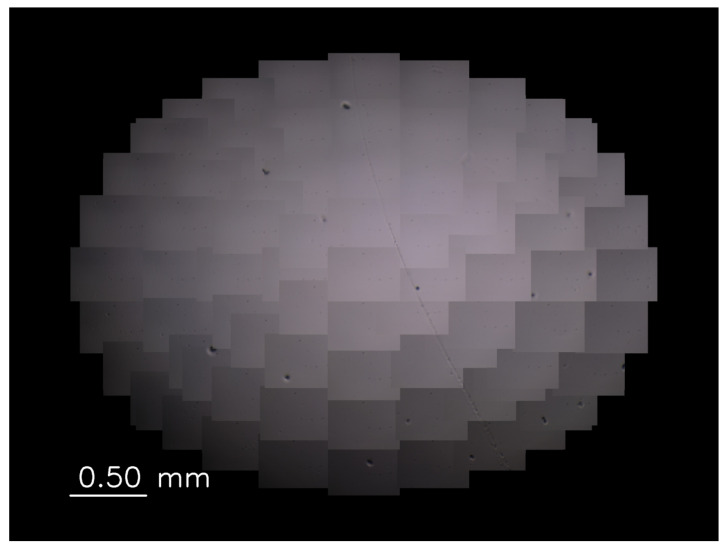
Magnified image without gamma correction.

**Figure 4:**
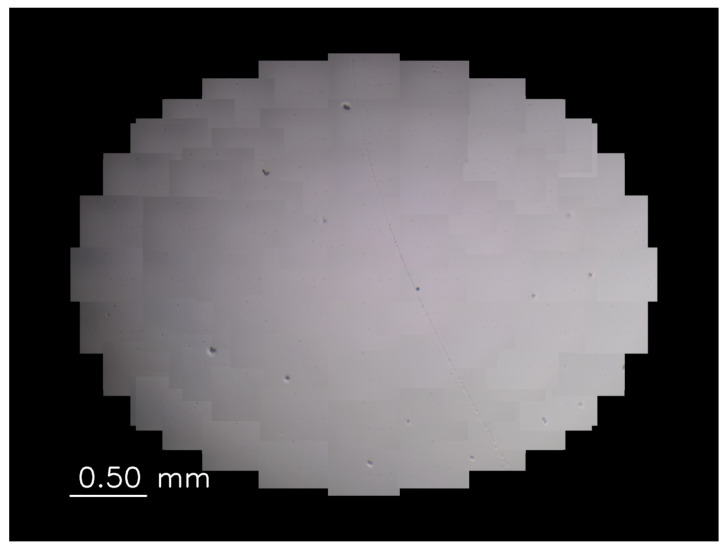
Magnified image with gamma correction.

Gamma correction is performed based on the gamma value set according to the mirror angle for each image from each viewpoint to ensure that the luminance of the wide-range images is bright and uniform. In this system, since the light intensity differs for each viewpoint, we experimentally derived the appropriate γ value at each viewpoint. Figure 3 and Figure 4, respectively, show the magnified images with and without gamma correction. The average luminance of Figure 3 is 93.9 and standard deviation is 32.0. On the other hand, the average luminance of Figure 4 is 146.2 and standard deviation is 31.3. The average luminance values increase while the standard deviations remain almost the same, indicating that the gamma correction is effective for obtaining bright and uniform magnified images.

The *n*th input image at time *t*, I(x,y,t,n) is gamma corrected with the value of γ(n), and the output image O(x,y,t,n) is derived as follows:

(1) $$\begin{array}{c} O\left(x,y,t,n\right)=255\times {\left(I\left(x,y,t,n\right)/255\right)}^{1/\gamma (n)}. \end{array}$$

(3) Binarization 

Binarization and combining of images from each viewpoint were performed using the threshold value set according to the angle of the mirror. A suitable threshold value for the *n*th input image T(n) is set according to these viewpoints.

The *n*th input image I(x,y,t,n) at time *t* is converted to a binarized image B(x,y,t,n) with the threshold value T(n):

(2) $$\begin{array}{c} B\left(x,y,t,n\right)=\left\{\begin{array}{c} 1,\left(I\left(x,y,t,n\right)\leq T\left(n\right)\right)\\ 0,\left(\mathrm{otherwise}\right) \end{array}\right.. \end{array}$$

(4) Connected-component labeling 

Connected-component labeling is performed to detect the self-propelled droplets in the binarized view-expansive images and to obtain their features. Since the view-expansive images have large image sizes, the scan plus array-based union-find (SAUF) algorithm is used for connected-component labeling, as it is known to be an efficient algorithm [17].

(5) Generation of the region of interest

A region of interest (ROI) is defined for the detected self-propelled droplets.

(i) Deviation of the center of gravity (CoG)

The 0th and 1st moments of a tracking target are calculated as follows:

(3) $$\begin{array}{ccc} \;\;\;\;\;\;\;\;\;\;\;\; & & M_{0}=\sum_{x,y}B\left(x,y,t\right), \end{array}$$

(4) $$\begin{array}{ccc} \;\;\;\;\;\;\;\;\;\;\;\; & & \;M_{x}=\sum_{x,y}xB\left(x,y,t\right),\;M_{y}=\sum_{x,y}yB\left(x,y,t\right). \end{array}$$

Then, the CoG of the tracking target $\left(c_{x},c_{y}\right)$ is derived as follows:

(5) $$\begin{array}{c} \left(c_{x},c_{y}\right)=\left(\frac{M_{x}}{M_{0}},\frac{M_{y}}{M_{0}}\right). \end{array}$$

Note that if $M_{0}$ is less than the threshold value $S_{0}$, the detected objects are removed as noise.

(ii) Definition of the size of the ROI 

The height and width of the ROI, i.e., height and width are respectively derived by calculating the coordinates of the top, bottom, left, and right edges of the detected self-propelled droplet area.

(6) $$\begin{array}{c} width=x_{max}-x_{min} \end{array}$$

(7) $$\begin{array}{c} height=y_{max}-y_{min}, \end{array}$$

where $x_{max}$, $x_{min}$, $y_{max}$, and $y_{min}$ are the coordinates of the top, bottom, left, and right edges, respectively.

Then, the ROI is defined using the larger of the vertical and horizontal widths as *L* on each side and the CoG as the center of the ROI.

(8) $$\begin{array}{c} L=\left\{\begin{array}{c} height,\left(width\leq height\right)\\ width,\left(\mathrm{otherwise}\right) \end{array}\right.. \end{array}$$

(6) Position detection 

Information regarding the position of the self-propelled droplets is obtained from the ROI. The position and color details of the self-propelled droplets in each frame are related between consecutive frames; the position and color information can be acquired as the time-series data of each self-propelled droplet.

First, the position information of the $N_{t-1}$ self-propelled droplets in frame t−1 is recorded as $p_{t-1}\left(1\right)∼p_{t-1}\left(N_{t-1}\right)$, and the position information of the $N_{t}$ self-propelled droplets in frame *t* is recorded as $p_{t}\left(1\right)∼p_{t}\left(N_{t}\right)$. Next, the position information of the self-propelled droplets in frames *t* and t−1 is compared; the closest positions between the droplets in the two frames $n_{t,t-1}\left(1\right)∼n_{t,t-1}\left(N_{t}\right)$ are obtained, and each droplet is linked to the same self-propelled droplet.

(9) $$\begin{array}{c} n_{t,t-1}\left(1\right)=\left\{\begin{array}{c} p_{t-1}\left(1\right),(|p_{t}\left(1\right)-p_{t-1}\left(1\right)\left|=min\right)\\ p_{t-1}\left(2\right),(|p_{t}\left(1\right)-p_{t-1}\left(2\right)\left|=min\right)\\ \vdots \\ p_{t-1}\left(N_{t-1}\right),(|p_{t}\left(1\right)-p_{t-1}\left(N_{t-1}\right)\left|=min\right) \end{array}\right. \end{array}$$

(10) $$\begin{array}{c} n_{t,t-1}\left(2\right)=\left\{\begin{array}{c} p_{t-1}\left(1\right),(|p_{t}\left(2\right)-p_{t-1}\left(1\right)\left|=min\right)\\ p_{t-1}\left(2\right),(|p_{t}\left(2\right)-p_{t-1}\left(2\right)\left|=min\right)\\ \vdots \\ p_{t-1}\left(N_{t-1}\right),(|p_{t}\left(2\right)-p_{t-1}\left(N_{t-1}\right)\left|=min\right) \end{array}\right. \end{array}$$

$$\begin{array}{c} \vdots \end{array}$$

(11) $$\begin{array}{c} n_{t,t-1}\left(N_{t}\right)=\left\{\begin{array}{c} p_{t-1}\left(1\right),\left(|p_{t}\left(N_{t}\right)-p_{t-1}\left(1\right)|=min\right)\\ p_{t-1}\left(2\right),\left(|p_{t}\left(N_{t}\right)-p_{t-1}\left(2\right)|=min\right)\\ \vdots \\ p_{t-1}\left(N_{t-1}\right),\left(|p_{t}\left(N_{t}\right)-p_{t-1}\left(N_{t-1}\right)|=min\right) \end{array}\right. \end{array}$$

To account for the possibility that the self-propelled droplets may be out of the imaging range, the droplets are correspondingly linked only when the distance between two frames of the self-driven droplets is less than the threshold value. In addition, a simple and stable method was adopted in this study as the speed of the self-propelled droplets is low, and the image processing speed is sufficient for an image acquisition rate of 1 fps.

(7) Acquisition of color information 

**Figure 5:**
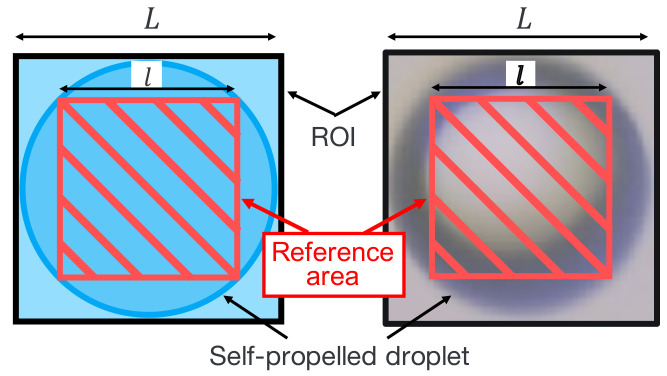
Reference area for color information acquisition.

Color information inside the self-propelled droplets is acquired to obtain the chemical reaction information of the droplets. The diameter of a self-propelled droplet *r* is estimated from the size of the ROI *L*. The region in which the color information is acquired is set as a square of sides of length *l*, as shown in Figure 5.

(12) $$\begin{array}{c} l=\sqrt{2}L \end{array}$$

The color information inside the self-propelled droplets is given by the average RGB value within the square with a side length of *l*.

(8) Color discrimination of self-propelled droplets 

In this study, the droplets were imaged in the oxidized and reduced states in advance, and a similarity evaluation was performed on the droplets in the acquired images to identify their colors. Since this system is based on bright-field observations, where the specimen is uniformly illuminated, a normalized cross-correlation function was used in the similarity evaluations.

First, the color histograms of the three channels of RGB values of the two images to be compared (A and B) are obtained, and the edge values of each bin of the two-color histograms are obtained. Let $H_{A}\left(i\right)$ and $H_{B}\left(i\right)$ be the edges of the two-color histograms in bin *i*. $H_{A}\left(i\right)$ and $H_{B}\left(i\right)$ are divided by the total number of bins in the color histogram *p*, and the normalized edges $h_{A}\left(i\right),h_{B}\left(i\right)$ are obtained.

(13) $$\begin{array}{c} h_{A}\left(i\right)=\frac{H_{A}\left(i\right)}{p} \end{array}$$

(14) $$\begin{array}{c} h_{B}\left(i\right)=\frac{H_{B}\left(i\right)}{p} \end{array}$$

We also derive the averages $\bar{h_{A}}$ and $\bar{h_{B}}$ for each of the bins of $h_{A}\left(i\right),h_{B}\left(i\right)$:

(15) $$\begin{array}{c} \bar{h_{A}}=\frac{1}{p}\sum_{i=0}^{p}h_{A}\left(i\right) \end{array}$$

(16) $$\begin{array}{c} \bar{h_{B}}=\frac{1}{p}\sum_{i=0}^{p}h_{B}\left(i\right) \end{array}$$

Then, the similarity s(A,B) between the bins is obtained by determining the correlation coefficient based on $\bar{h_{A}}$ and $\bar{h_{B}}$:

(17) $$\begin{array}{c} s\left(A,B\right)=\frac{\sum_{i=0}^{p}\left(h_{A}\left(i\right)-\bar{h_{A}}\right)\left(h_{B}\left(i\right)-\bar{h_{B}}\right)}{\sqrt{\sum_{i=0}^{p}{\left(h_{A}\left(i\right)-\bar{h_{A}}\right)}^{2}\sum_{i=0}^{p}{\left(h_{B}\left(i\right)-\bar{h_{B}}\right)}^{2}}} \end{array}$$

Similarities are derived for each of the three channels of RGB values, and the average value is used to identify the color of the droplet. Note that the similarity s(A,B) is given by −1≤s(A,B)≤1.

## 3. Experiments

### 3.1. Experimental Condition

**Figure 6:**
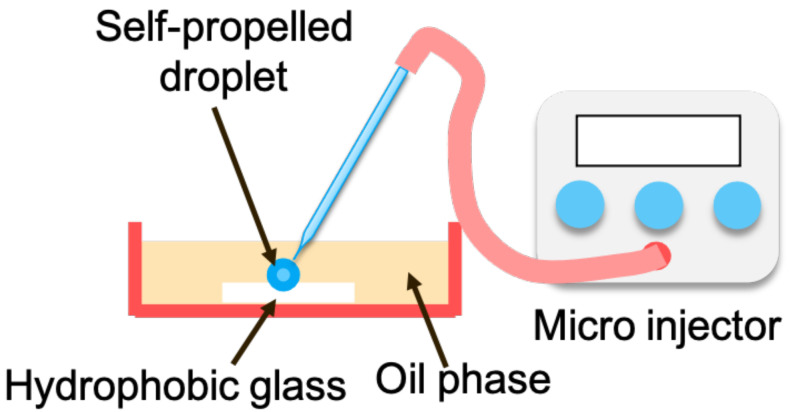
Experimental condition.

Figure 6 shows a schematic of the experimental environment. A slide glass with a hydrophobic coating was placed at the bottom of a petri dish, and a cellophane partition was placed on the slide glass. The petri dish was filled with a monoolein-squalene solution (10 mM). Multiple self-propelled droplets were generated on the hydrophobically coated slide glass using a microinjector, and image analysis of the self-driven droplets was conducted in these experiments. Because the water droplets have a higher specific gravity than oil, the droplets sink and move autonomously within the partition.

### 3.2. Tracking Experiment for Self-Propelled Droplets

We conducted a tracking experiment on multiple self-propelled droplets to verify the tracking function of the proposed system. Droplets were generated by injecting water droplets in an ambient solution of monoolein-squalene (10 mM). The self-propelled droplets were a mixture of sulfuric acid (3.0 M), sodium bromate (2.0 M), and reaction indicator ferroin (20 mM) in a 1:1:1 ratio. Ferroin was obtained through the following chemical reaction: 1,10-phenanthroline was mixed with iron (II) sulfate heptahydrate.

(18) $$Fe^{2+}+3\mathrm{phen}\to {\left[Fe\left(\mathrm{phen}\right)3\right]}^{2+}$$

**Figure 7:**
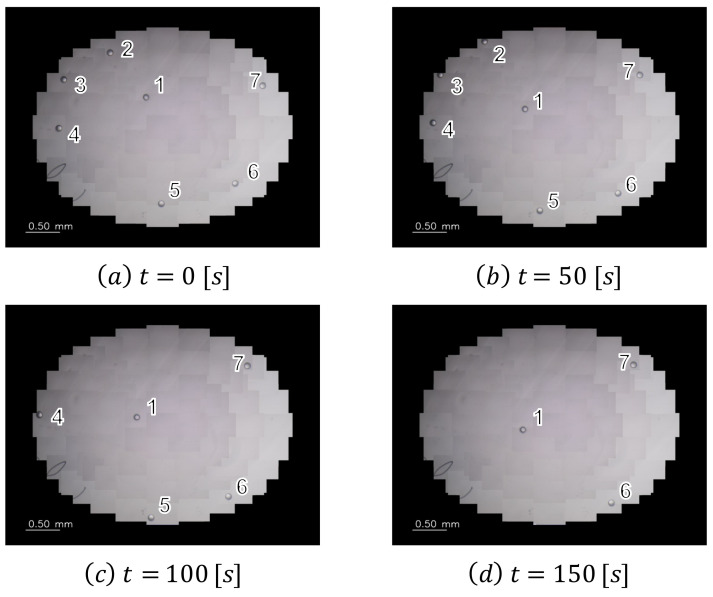
Large field-of-view images.

**Figure 8:**
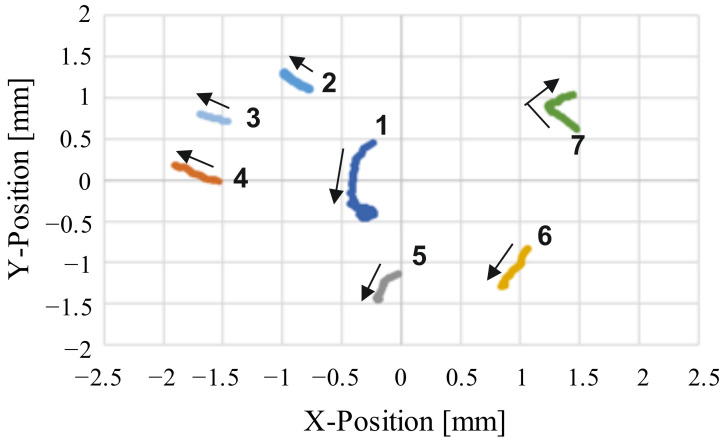
Changes in the positions of the droplets over time.

In the verification experiment, the imaging range of the wide-range image was set to an elliptical region of 3.8 × 2.9 mm, the frame rate was set to 1.0 fps, and the motions of the self-driven droplets were recorded for approximately 17 min. Figure 7 shows snapshots of the captured images, and Figure 8 shows the trajectories of the self-driven droplets. The origin of the coordinate system is the center of the wide-range image. In this experiment, the system was able to track the droplets without interruptions for 17 min, during which the experiments were conducted. These results confirm that the proposed system can track multiple self-driven droplets. The proposed visual sensing system can detect self-propelled droplets from a wide range of high-resolution images and analyzes spontaneous movements through a motion tracking algorithm for view-expansive images.

### 3.3. Color Identification of Self-Propelled Droplets

To verify the color identification function of the proposed system, we conducted a color analysis experiment on blue and red self-propelled droplets.

The droplets were generated by injecting water droplets in an ambient solution of monoolein-squalene (10 mM). The blue droplets consisted of sulfuric acid (3.0 M), bromine (2.0 M), and ferroin (20 mM), while the red droplets consisted of the reaction indicator ferroin (20 mM). The generated self-propelled droplets moved spontaneously on a cover glass with a hydrophobic coating inside the petri dish.

**Figure 9:**
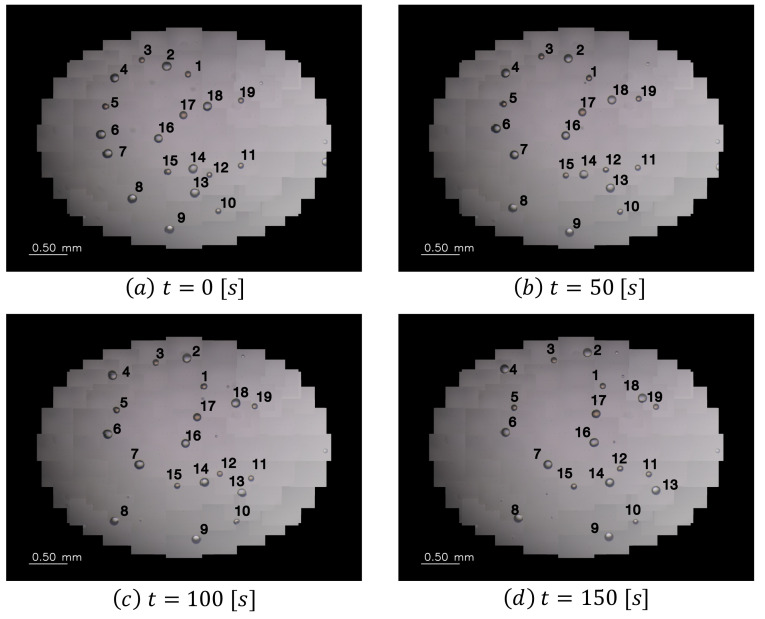
Large field-of-view images.

**Figure 10:**
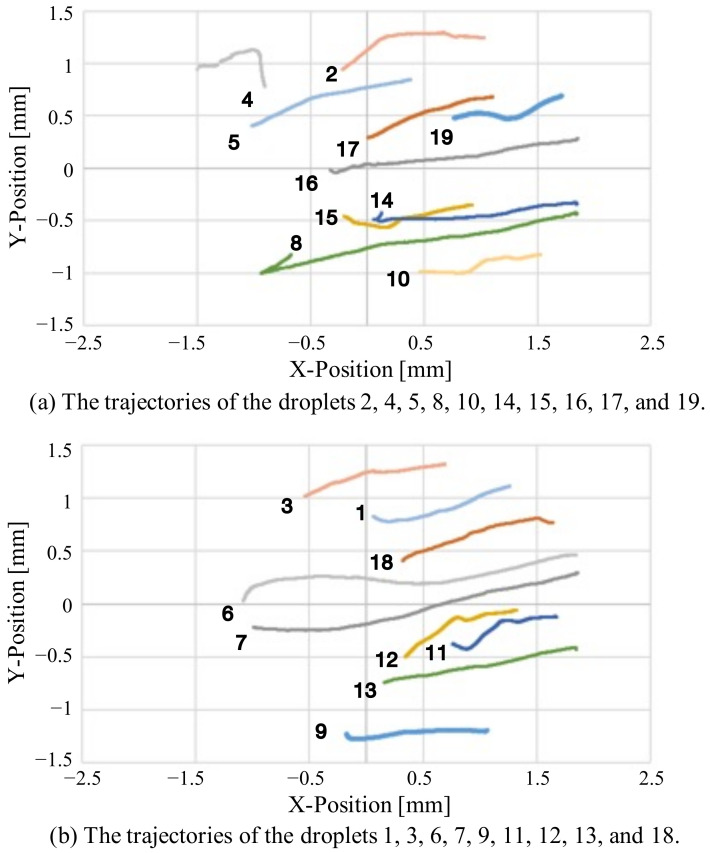
Changes in the position of a droplet over time.

In this experiment, the imaging range of the wide-range image was set to an elliptical region of 3.8 × 2.9 mm, frame rate was set to 1.0 fps, and motions of the self-driven droplets were recorded for approximately 20 min. Figure 9 shows snapshots of the captured images, and Figure 10 shows the trajectory of the self-driven droplets. For visibility, the trajectories have been split into two graphs. The origin of the coordinate system is the center of the wide-range image. These results confirm that the proposed system can track self-driven droplets of multiple colors.

**Figure 11:**
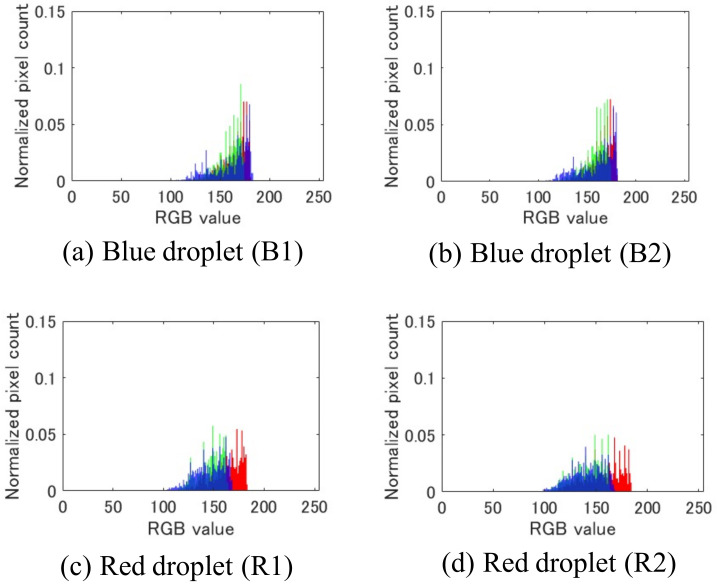
Color histograms of droplets. The red bars represent the red histogram, the green bars represent the green histogram, and the blue bars represent the blue histogram.

Figure 11 shows the histograms of two blue droplets (B1 and B2) and two red droplets (R1 and R2) obtained using the proposed system. From these histograms, the similarity calculation in Equation (17) yields the following results:

(19) $$\begin{array}{c} s(B1,B2)=0.96 \end{array}$$

(20) $$\begin{array}{c} s(R1,R2)=0.91 \end{array}$$

(21) $$\begin{array}{c} s(B1,R1)=0.52 \end{array}$$

(22) $$\begin{array}{c} s(B2,R2)=0.47 \end{array}$$

The results demonstrate that the similarity between droplets of the same color is more than 0.9, while the similarity between droplets of different colors is approximately 0.5, indicating that the similarity between droplets of the same and different colors is clearly different. Hence, the image resolution of the proposed system is sufficient for color discrimination, which can be used to determine the chemical reactions of the droplets. The proposed visual sensing system can identify the internal colors of the droplets for analysis of the chemical reactions through a color camera head and color identification algorithm.

## 4. Conclusions

In this study, we propose an image sensing system that detects self-propelled droplets from a wide range of high-resolution images and analyzes their spontaneous motions and chemical reactions. It is typically difficult to analyze both the motions and chemical reactions of the self-propelled droplets using conventional sensing systems. Our proposed visual sensing system enables both wide-range imaging for analyzing the interactions between multiple droplets and high-resolution imaging for observation of the chemical reactions. The effectiveness of this system is confirmed through motion-tracking and internal color identification experiments on the self-propelled droplets. The proposed method for analyzing images of a view-expansive microscope can be applied to create chemical measures and be used in automatic sensors of the collective self-propelled droplets. In a future study, it is expected that a detailed analysis of the self-propelled droplets with autonomous motions using the Belousova-Jabotinsky reaction will be conducted. If the micrometer-sized self-propelled droplets can be controlled through further studies, it is expected that this knowledge can be applied to drug delivery systems.

## Data Availability

Not applicable.
